# 右肺上叶、中纵隔并发成熟性畸胎瘤1例

**DOI:** 10.3779/j.issn.1009-3419.2011.05.15

**Published:** 2011-05-20

**Authors:** 水洪 蓝, 新华 王, 君峰 陈

**Affiliations:** 1 311502 桐庐，浙江省桐庐县第三人民医院 The Third People's Hospital of Zhejiang Province Tonglu County, Tonglu 311502, China; 2 310006 杭州，浙江中医药大学附属第一临床医学院呼吸科 Department of Respiratory, the First Clinical Medical School Affiliated to Zhejiang Chinese Medical University, Hangzhou 310006, China; 3 310053 杭州，浙江中医药大学硕士研究生 Zhejiang Chinese Medical University, Hangzhou 310053, China

纵隔畸胎瘤是纵隔肿瘤中较常见的肿瘤之一，发生率约占原发性纵隔肿瘤的21.5%^[1]^，而发生在肺部的成熟性畸胎瘤（intrapulmonary matureteratoma, IPT）较为罕见，1839年由Mohr首次报道^[2]^。迄今国内外也仅有散在个例报道^[3, 4]^，而在肺内、纵隔并发的畸胎瘤更鲜有报道，现将浙江省桐庐县第三人民医院已确诊的1例肺内、纵隔并发的成熟性畸胎瘤报道如下。

## 1 临床资料

患者，女性，37岁，因“发热、咳嗽伴右侧胸痛一月余”入院。于1个月前在无明显诱因下出现畏寒、高热，体温最高时达39.5 ℃，咳嗽阵作，咳痰色黄，量不多，无腥臭味。自服感冒药及抗炎药，症状未缓解，高热持续，出现右侧持续性胸痛。十余天后至当地医院住院治疗。查血常规：WBC：12.3×10^9^/L，NE%：83.6%。2月11日胸部CT示（图 1A、图 1B）右上肺可见两个相连的、大小约5.0 cm×5.0 cm的肿块，右下肺巨大肿块，大小约10.0 cm×10.0 cm，肿块密度低，边有包囊，诊断为右肺脓肿。先后予左氧氟沙星及替硝唑、注射用头孢哌酮钠舒巴坦钠抗感染，盐酸氨溴索化痰及营养支持等对症治疗。半月前行“右下肺脓肿”穿刺引流，共抽出黄色豆腐渣样浑浊液体约350 mL，抽出物送检显示CEA：545.78ng/L，CA199 > 1, 000 u/L。后患者高热渐退，体温降至正常，咳嗽、咳痰明显减少，胸痛缓解，体重有所减轻。复查胸部CT示右下肺肿块明显减小，右上肺尖后段肿块仍存在，为进一步治疗于3月3日入住我院。体检：右肺语颤减弱，上、下部叩诊音浊，右肺呼吸音偏低，左肺呼吸音清，两肺未及明显干湿啰音。

**1:**
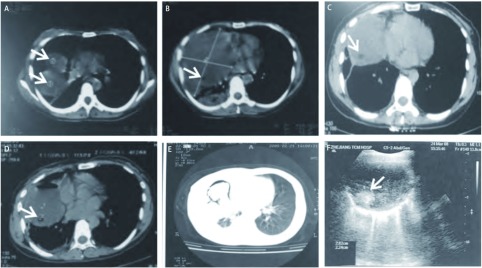
胸部CT及B超所见。A：（2月11日）右上叶肿块与纵膈成锐角，中间有肺组织分膈，肿块中间有高密度影（箭头所指）；B：（2月11日）右下块纵膈紧密相连，没有含气组织及脂肪间膈，且与纵膈呈钝角，肿块中间有低密度影（箭头所指）；C（3月5日）、D（3月24日）：右下肺肿块内始终存在一类圆形低密度影（箭头所指），CT值：- 61.2 Hu；E：液体抽取后，出现气体和固态物质夹杂的征象；F：B超检查示囊性病灶中可见一类园形高回声影，边界清楚（箭头所指） The appearance of chest CT and B ultrasound. A: (11th, February) there is a tumor which high density image inside and devided by lung tissue in right upper lobes (arrow); B: (11th, February) a tumor which low density image inside and without gas and fat tissue (arrow); C (5th, March), D (24th, March): a tumor which spherical low density image inside in right lower lobes (arrow), CT value: -61.2 Hu; E: After draw fluid, there appear gas and solid matter inclusion image; F: B ultrasound show there is a hyperechogenicity image in a cystica tumor (arrow)

入院予注射用头孢哌酮钠舒巴坦钠抗感染以及营养支持等对症治疗及完善相关辅助检查：血常规：WBC：5.3×10^9^/L，NE%：59.10%，CRP：6 mg/L；血肿瘤类：CEA：3.4 ng/mL。3月17日B超定位后再次行右下肺肿块穿刺抽液引流术，抽出少量褐色豆腐渣样浑浊液体并送检示：胸水常规细胞破坏明显；ADA：134.0 u/L，LDH：8, 910.0 IU/L，CEA：2, 900.0 ng/mL，CA19-9：385, 773.0 u/L。此时患者一般情况较可，没有发热、咳嗽、咳痰等任何不适症状。3月5日和3月24日胸部CT复查发现右下肺肿块内始终存在一类圆形低密度影，CT值：-61.2 Hu（图 1C、图 1D）。液体抽取后，出现气体和固态物质夹杂的CT征象（图 1E）。而B超亦提示一类圆形高回声团，边界清楚（图 1F）。遂于3月19日在B超引导下行经皮穿刺活检，穿刺物送检病理提示为少量纤维、脂肪组织和皮脂腺汗腺等组织。3月24日邀胸外科会诊后转胸外科治疗，完善各项术前准备，于4月1日在全麻下行右胸肿块切除术，术后患者恢复良好。

## 2 病理检查

### 2.1 巨检结果

术中见右侧全胸广泛粘连，部分致密，叶间裂疏松粘连，探查右上叶，脏层胸膜下有一肿块直径约6 cm×6 cm×4 cm（图 2A），肿瘤周围可见受压迫肺组织，沿包膜完整切除肿块，肿块质韧，呈囊性，囊壁厚0.4 cm-0.8 cm，囊内壁灰褐色，较光滑，内为皮脂样物（图 2B），病理报告为囊性成熟性畸胎瘤。右中纵隔肿块，直径约10 cm×10 cm×8 cm，肿块与上下叶无粘连侵犯，于中叶粘连致密，肿块性质同前。

**2:**
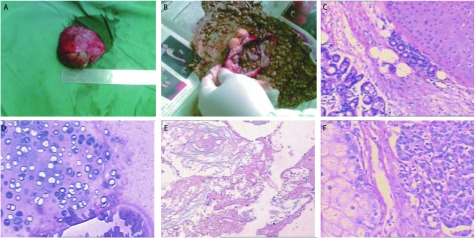
大体标本及镜下所见。A：右肺上叶肿块，直径约6 cm×6 cm×4 cm；B：肿块呈囊性，囊内为皮脂样物；C、D、E：右肺上叶肿块病理显示混合腺、软骨及角化物（HE, ×200）；F：右中纵膈肿块病理显示皮脂腺和胰腺组织（HE, ×200） The appearance of roughly speciem and under microscope. A: a tumor in right upper lobes, diameter 6 cm×6 cm×4 cm; B: cystica tumor, sebum cutaneum inside; C, D, E: tumor pathology show mixed gland, cartilage and keratosis(HE, ×200); F: tumor pathology show sebaceous glands and pancreatic gland tissue (HE, ×200)

### 2.2 镜检结果

肿瘤包含三个胚层来源的组织和器官样结构，主要由外胚层和内胚层成分构成，各组织分化成熟。右肺上叶囊壁由复层扁平上皮和假复层纤毛柱状上皮交替衬附，其内可见皮脂腺（图 2C）、汗腺、毛囊、软骨（图 2D）及浆粘液样腺体，伴有成片淋巴细胞、浆细胞浸润，肿瘤周围为厚薄不均的纤维包绕并过渡为受挤压的肺组织（图 2E），右中纵隔肿瘤内可见脂肪及胰腺组织（图 2F）。

### 2.3 病理诊断

右中纵隔并右肺内囊性良性畸胎瘤。

## 3 讨论

### 3.1 文献复习

畸胎瘤是一种生殖细胞来源的肿瘤，含有3个胚层演化的多种组织成分，好发于性腺及身体的中轴、中轴旁部位，最常发生于卵巢、睾丸，亦可发生于纵隔、腹膜后、肠系膜、骶尾部、颈、松果体等，发生于肺部者较罕见^[5]^，而在肺内、纵隔并发的畸胎瘤更为罕见。

纵隔畸胎瘤的发生，一般认为是由于胚胎时期第3、4对鳃弓发育异常而致，主要为部分多潜能组织，并随心血管的发育进入纵隔所致^[6]^。纵隔畸胎类肿瘤多见于前、中纵隔，肿瘤边缘多较光滑，少有分叶，密度多较均匀，边缘偶有钙化，囊实性或实性畸胎瘤多为混杂密度，如有脂肪液平面时，对诊断良性畸胎瘤有特异性。肿瘤内如有牙齿或骨骼亦为畸胎瘤的特征表现。而肺内畸胎瘤的发生与胸腺的始基第3对咽囊有关^[7]^。肺内畸胎瘤被证明是各胚层未分化的迷走胚胎性组织沿支气管下行，为肺胚基包绕形成的肿瘤。肺内畸胎瘤和纵隔畸胎瘤都有典型的畸胎瘤样改变，多为良性，实质性肿瘤小部分为恶性。肿瘤由外胚层衍化而来例如皮肤、毛发和皮肤附属器，囊肿部分由鳞状上皮衍化而来，衬里含有浓缩皮脂物质。任何三胚层起源的组织在肺内畸胎瘤内均可出现。显微镜下囊壁可覆有典型皮肤、皮脂腺、支气管壁等结构，个别报道有胸腺组织。肺内畸胎瘤的病变部位曾认为好发于左上叶前段^[8]^，但综合病例统计显示，两肺上叶前段（包括左舌上段）均好发，较少发生在右中叶和两肺下叶，可跨叶生长^[8]^。

肺内畸胎瘤与纵隔畸胎瘤在临床上一般较难鉴别，患者一般症状较轻或仅有轻度胸闷等症状，部分患者在体检时发现，常在21岁-40岁年龄段诊断，多因并发症如感染、支气管扩张等就医。最常见症状依次为胸痛、发热、咳嗽、咳痰和咳血^[8]^。咳出毛发和皮脂样物提示肿瘤与气道相通。若合并感染常以肺脓肿、肺炎、肺不张为主要特征。X线上见胸前部肿块，如证实病变内含钙化（特别是齿形或弧线形钙化）、脂肪和软组织等多种成分提示畸胎瘤诊断，但须确定肿块位于肺内还是纵隔。CT上一般有以下几点可鉴别：①肿块与纵隔相交角度：锐角多为肺内，钝角多位肺外；②胸部CT纵隔脂肪间隙存在：提示肺内肿块，不是纵隔肿块向肺内延伸；③畸胎瘤肿块周边部含气透光（类似“空气新月”征）：表示肿瘤原发于肺或支气管腔内，亦有可能是畸胎瘤破裂与气道交通^[9]^。该病例右肺上叶肿块与纵隔呈锐角，肿块与之间有含气肺组织，提示肿块位于肺内，即右肺上叶肿块为肺内畸胎瘤。而右下肿块纵隔紧密相连，没有含气组织及脂肪间歇，且与纵隔呈钝角，提示肿块位于纵隔，即右下肿块为中纵隔畸胎瘤。

对于肺内、纵隔畸胎瘤的治疗目前主要采取手术切除病变部位的方法，术后患者预后好。有研究^[10]^报道1例恶性畸胎瘤者手术切除后17年仍存活良好。若不及时手术切除肿瘤，患者可因严重感染、大咯血或肿瘤恶变（仅占1%）致死^[11]^。

### 3.2 经验分析

本例右肺上叶并中纵隔畸胎瘤，最初误诊为右肺脓肿，最后经B超定位下经皮肺穿、剖胸探查切除、病理检查，得以确诊为肺内、纵隔畸胎瘤继发脓肿样病变。作者认为其原因如下：①临床表现为发热、咳嗽、气促明显，有白细胞总数及中性粒细胞升高等感染征象；②肺部体征如叩诊右肺呈浊音，右肺呼吸音减弱等；③初次胸片、CT、B超检查胸部均提示右上肺和右下肺巨大脓肿；④抗生素治疗后临床症状一度明显改善，没有发热、咳嗽、咳痰等任何不适症状，从而导致作出肺脓肿之错误诊断。但是仔细分析临床病例资料，作者发现：①先后两次经胸导管抽取近450 mL黄油状液体，外观混浊，夹杂有黄色豆腐渣样坏死组织，这与常见的肺部脓肿引流液性质不同；②送抽取液常规细胞破坏明显，以致无法做常规检查，且多次送培养均无细菌生长；③液体抽取后，出现气体和固态物质夹杂的CT征象（图 1E），且液体生长速度快，很快就将囊腔填满；④胸部CT示右上肺肿块内有高密度影，而右下肺囊性病灶里有极低脂肪密度影，B超检查于囊性病灶中可见一类圆形高回声影，边界清楚。以上诸点均提示不能单纯考虑肺部脓肿，特别是最后一点，作者在B超医生作囊腔穿刺定位时提出类圆形高回声影为何物的疑问，在B超引导下经皮穿刺此高回声影，获得病理结果为畸胎瘤，最后剖胸切除，提示临床医生需时刻关注患者出现的每个细节，这往往是疾病诊断的关键。

特别要指出的是，该病例曾一度考虑是否为恶性肿瘤，因其抽出物液多次送检肿瘤指标物CEA持续增高，最高时CEA达2, 900.0 ng/mL，但最后确诊为肺内及纵隔良性畸胎瘤，其增高原因可能为继发脓肿。有文献^[12]^报道脓液中CEA明显升高，这也给我们一个有益的启迪，即：临床工作不能仅靠单个指标来考虑问题，有疑问时还需查阅相关文献资料，不断积累临床经验。此外，该病例抽出物液中ADA、CA19-9亦明显增高，是否与脓性感染有关值得我们进一步研究。
